# Beyond the Warburg Effect: How Do Cancer Cells Regulate One-Carbon Metabolism?

**DOI:** 10.3389/fcell.2018.00090

**Published:** 2018-08-15

**Authors:** Adam Rosenzweig, John Blenis, Ana P. Gomes

**Affiliations:** ^1^Meyer Cancer Center, Weill Cornell Medicine, New York, NY, United States; ^2^Department of Pharmacology, Weill Cornell Medicine, New York, NY, United States

**Keywords:** one carbon metabolism, cancer, metabolic reprogramming, folate cycle, methionine cycle, metabolic regulation

## Abstract

Altered metabolism in cancer cells is critical for tumor growth. One of the most notable aspects of this metabolic reprogramming lies in one-carbon metabolism. Cells require one-carbon units for nucleotide synthesis, methylation reactions, and for the generation of reducing cofactors. Therefore, the ability to rewire and fine-tune one-carbon metabolism is essential for the maintenance of cellular homeostasis. In this review, we describe how the major nutrient, energy, and redox sensors of the cell play a significant role in the regulation of flux through one-carbon metabolism to enable cell fate decisions. We will also discuss how dysregulated oncogenic signaling hijacks these regulatory mechanisms to support and sustain high rates of proliferation and cell survival essential for tumor growth.

## Introduction

One-carbon metabolism encompasses a broad range of biosynthetic reactions that occur in the cytoplasm and the mitochondria which are essential for maintaining cellular homeostasis. These pathways catabolize different carbon sources to derive one-carbon (methyl) units to be utilized in fundamental cellular functions (Ducker and Rabinowitz, 2017). Due to the specific manner in which one-carbon units are obtained and utilized, one-carbon metabolism serves as an integrative pathway, relating many nutrients to one another. Flux through one-carbon metabolism must remain plastic for cells to regulate levels of the related nutrients in response to ever-changing intra- and extracellular conditions. One-carbon metabolism provides cells with the building blocks, as well as the reducing power, necessary to maintain high rates of proliferation, and therefore is key in supporting cancer. In this review, we discuss how cells regulate flux through one-carbon metabolism and its implications for tumorigenesis.

## One-carbon metabolism, integrator of nutrient status?

One-carbon metabolism is essential in cellular physiology as it functions as an integrator of the nutritional status of cells. One-carbon units are derived from different nutrients inputs and generate various molecular outputs that serve as building blocks for biosynthesis, methylation and redox reactions.

One-carbon units are largely derived from the non-essential amino acids serine and glycine (Kalhan and Hanson, 2012). Both serine and glycine can be obtained exogenously or synthesized from other carbon sources. Serine can be created *de novo* from glucose through a series of enzymes which convert 3-phosphoglycerate (3PG) into serine [phosphoglycerate dehydrogenase (PHDGH), phosphoserine aminotransferase 1 (PSAT1), and phosphoserine phosphatase (PSPH) –referred hereafter as the serine synthesis pathway (SSP)] (Locasale, 2013). Glycine can be produced from serine or threonine (Wang et al., 2009). Hypothetically, both serine and glycine can equally donate one-carbon groups, however, the actual contribution of serine and glycine is far more complex. Serine is believed to be the more significant one-carbon unit donor, but glycine can also contribute one-carbon units through oxidation by the glycine cleavage system (GCS), though in lesser quantities than the serine-to-glycine conversion (Tedeschi et al., 2013). It is clear that glycine catabolism is important for one-carbon metabolism, however the relative contribution of glycine vs. serine is still debated. Several reports showed that cancer cells fail to consume glycine when serine is abundant (Maddocks et al., 2013; Labuschagne et al., 2014), while others have shown a significant upregulation in glycine consumption (Jain et al., 2012). It is likely that the relative contribution of either serine or glycine to fuel one-carbon metabolism is dependent on cell type and environment.

**Figure 1:**
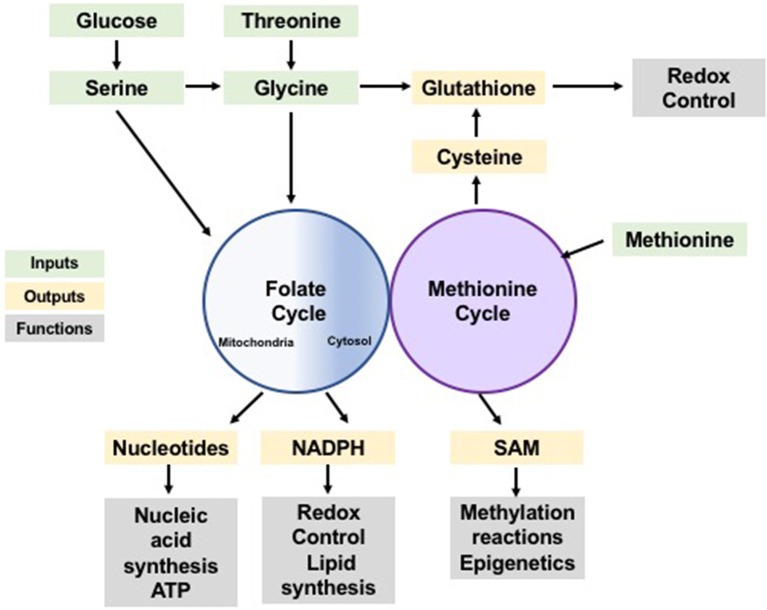
One-carbon metabolism as a cellular process integrating nutrient status and availability. Glucose and amino acids input to the folate and methionine cycles (green) contributing with one-carbon units which can be used in anabolic synthesis of many building blocks, reducing species and co-factors (yellow). These synthesis products support a variety of cellular functions (gray) including synthesis of biomolecules, redox control and post-translational modification, sustaining cellular homeostasis.

One-carbon units are utilized in two pathways: the folate cycle and the methionine cycle (Figure 1). In the folate cycle, folic acid is reduced by dihydrofolate reductase (DHFR) to the biologically active tetrahydrofolate (THF) (Newman and Maddocks, 2017). In this reduced form, one-carbon units from serine and glycine can be transferred by serine hydroxymethyltransferase (SHMT) and glycine decarboxylase (GLDC; of the glycine cleavage system [GCS]), respectively, onto THF forming methyl-THF. Once methylated, THF can undergo a series of redox transformations by the multi-functional enzyme methylenetetrahydrofolate dehydrogenase (MTHFD1/2/1L), which has cytosolic and mitochondrial isoforms (Lewis et al., 2014; for detailed information on the folate cycle see Ducker and Rabinowitz, 2017). In the methionine cycle, homocysteine is re-methylated using a one-carbon unit from methyl-THF to form methionine via methionine synthase (MS) (Yang and Vousden, 2016). Demethylation of S-adenosyl-methionine (SAM) yields S-adenosyl-homocysteine (SAH), which is then converted to homocysteine, completing the cycle. The crosstalk between the folate and methionine cycles goes beyond the re-methylation of homocysteine, as *de novo* ATP synthesis powered by the folate cycle directly contributes to the formation of SAM, a critical donor to multiple methylation reactions (Maddocks et al., 2016). Furthermore, *de novo* generation of ATP by the folate cycle is likely to be indispensable in maintaining energetic homeostasis, particularly in conditions of high energy demand such as sustaining high rates of proliferation.

It is noteworthy that the folate cycle functions in the cytosol and in the mitochondria, although most proliferating cells rely primarily on the mitochondrial pathway (Tibbetts and Appling, 2010; Ducker et al., 2016). This compartmentalization is hypothesized to be a mechanism by which cells can decouple one-carbon metabolism from glycolysis (Ducker and Rabinowitz, 2017). One-carbon metabolism produces a large quantity of NADH, and localization to the mitochondria preserves cytosolic NAD^+^ which is necessary for glycolysis reactions (Ducker and Rabinowitz, 2017). The mitochondrial pathway may also convey an energetic advantage over the cytosolic. Evidence suggests that excess formate resulting from serine metabolism is exported from the mitochondria, and with each formate exported, an ADP molecule is phosphorylated to ATP (Meiser et al., 2016). Whether these two branches of the folate cycle are redundant or not is still controversial. Some groups have shown that the cytosolic folate cycle cannot compensate for loss of the mitochondrial pathway (Celardo et al., 2017), others have shown the opposite (Ducker et al., 2016). It is likely that the existence or the lack of redundancy between these arms of the folate cycle is also context dependent (Ducker et al., 2016).

Together the folate and methionine cycles mediate the redistribution of one-carbon groups derived from nutrients into the production of purine nucleotides (Tedeschi et al., 2013), glutathione, (Zhou et al., 2017), ATP, and NADPH; to control cell fate and maintain homeostasis (Tedeschi et al., 2013; Maddocks et al., 2016). Yet, the influence of one-carbon metabolism goes beyond energy currency and redox power. SAM plays a significant role in epigenetics, in post-translational modifications, and in signaling pathways through its contribution to methylation reactions (Finkelstein, 1990; Su et al., 2016). Therefore, one-carbon metabolism not only dispenses carbon atoms to various acceptor molecules, but it also integrates nutrient status with epigenetic, energetic, and redox statuses to maintain cellular homeostasis (Rowe and Lewis, 1973; Figure 1).

## Nutrient and energy sensors keep tabs on one-carbon metabolism

The mammalian target of rapamycin (mTOR) is a critical rheostat for the maintenance of metabolic balance and tightly regulates many aspects of metabolism (Gomes and Blenis, 2015). When nutrient availability is high, mTOR is activated, promoting anabolic reactions to sustain growth and proliferation. mTOR is a major regulator of one-carbon metabolism. One of the main effectors of mTOR for metabolic regulation is the activating transcription factor 4 (ATF4). When cellular serine levels are low, ATF4 is activated, leading to its binding to the promoter of the genes encoding SSP enzymes (Ye et al., 2012). This then drives their expression and consequentially increases serine pools (Ye et al., 2012). Moreover, mTOR signaling through ATF4 also regulates the expression of MTHFD2, stimulating the mitochondrial branch of the folate cycle (Ben-Sahra et al., 2016). Highlighting the importance of mTOR for one-carbon metabolism, another transcription factor that acts downstream of mTOR, FOXK1, is found to regulate the SSP, SHMT2, and MTHFD1L (He et al., 2018). As the forward flux of the folate cycle occurs in the mitochondria, the various forms of methylated THF are synthesized and then must be translocated to contribute to anabolism (Brosnan et al., 2015; Ducker et al., 2016). In the case of 10-formyl THF, it has been shown that the complex of enzymes which receive its one-carbon units, called the purinosome, is formed in the cytosol and colocalizes with the mitochondria under purine deficiency to expedite the synthesis of purine molecules (Zhao et al., 2013; Chan et al., 2015). This colocalization is thought to be mTOR dependent (French et al., 2016). Interestingly, one-carbon metabolism also signals back to mTOR as SAM was recently found to directly regulate mTORC1 activity (Gu et al., 2017).

One-carbon metabolism provides units to support growth and proliferation when nutrient availability and energy levels are high. Conversely, when these factors are limited, a brake is needed to slow down anabolic reactions and conserve energy. Such a mechanism relies on the activation of the AMP-activated kinase (AMPK). AMPK is the energy sensor of the cell; when ATP levels are high, AMPK is inhibited allowing anabolic reactions to transpire. When ATP levels are low and AMP levels rise, AMPK is activated, inhibiting anabolism and promoting catabolic reactions to restore ATP levels (Gomes and Blenis, 2015). Interestingly, AMPK has recently been shown to downregulate the expression of MTHFD1/2/1L through the PGC-1α/ERRα axis (Audet-Walsh et al., 2016). This suppression limits the flux of one-carbon units from serine and glycine to the products of one-carbon metabolism, thereby conserving energy.

**Figure 2:**
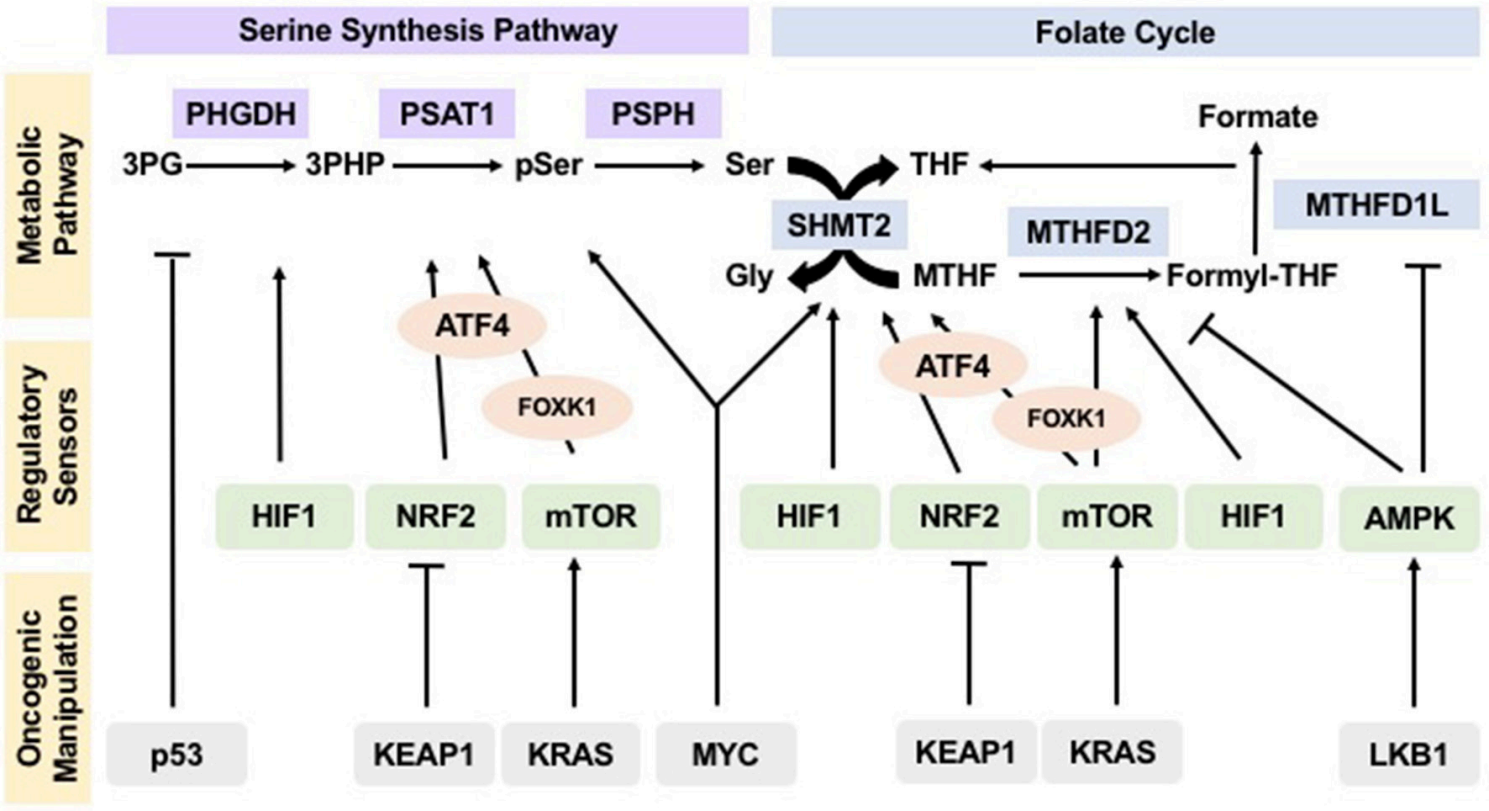
Oncogenes and tumor suppressors manipulate regulation of one-carbon metabolism pathways in cancer to drive tumorigenesis. Sensors of nutrient and energy levels, mTOR and AMPK, and sensors of redox potential, HIF1 and NRF2, regulate the different steps in one-carbon metabolism to ensure proper flux. In cancers, oncogenes KEAP1, KRAS, and MYC, as well as tumor suppressors p53 and LKB1, manipulate these regulatory sensors, thus affecting the operation and flux of pathways within one-carbon metabolism and allowing for their hyperactivity to sustain uncontrolled proliferation and tumorigenesis.

Together, these observations suggest that cells tightly regulate flux through one-carbon metabolism. This regulation is established based upon the energetic status of the cell and creates the metabolic flexibility necessary to maintain homeostasis (Figure 2).

## Redox and oxygen sensing mechanisms and one-carbon metabolism regulation

Under physiological conditions, the balance between generation and elimination of reactive oxygen species (ROS) maintains the proper function of redox sensitive pathways. When redox homeostasis is disturbed, oxidative stress contributes to disease development and can also lead to aberrant cell death. Therefore, eukaryotic cells have evolved systems to tightly regulate redox balance (Panieri and Santoro, 2016). Key components of these systems are the cofactors NADH and NADPH. NADPH plays a crucial role in the cell as it provides the reducing power that enables lipid synthesis and nucleotide synthesis, and the oxidation-reduction involved in detoxification of ROS (Panieri and Santoro, 2016). Recently, one-carbon metabolism has gained recognition as a main regulator of NADPH levels in the cells through the action of the MTHFD enzymes (Fan et al., 2014). Depletion of either the cytosolic or mitochondrial MTHFD enzymes resulted in decreased cellular NADPH/NADP+ and increased sensitivity to oxidative stress (Fan et al., 2014).

The nuclear factor erythroid-derived 2 (NRF2) coordinates an evolutionarily conserved transcriptional activation pathway that mediates antioxidant and detoxification responses, which is activated in response to oxidative stress. NRF2 has been shown to lead to the upregulation of the SSP and SHMT enzymes, through induction of ATF4 (DeNicola et al., 2015). This transcriptional upregulation works to promote serine flux through the folate cycle, consequently increasing the production of NADPH and powering cells with the reducing equivalents necessary to detoxify ROS (DeNicola et al., 2015). Moreover, the hypoxia inducible factor 1 (HIF1), which senses cellular oxygen levels, has also been shown to act as an activator of the SSP, mediating transcription of the genes involved in the SSP as well as SHMT2 (Iyer et al., 1998; Samanta et al., 2014). This allows the cells to build redox power to combat the build-up of ROS generated by hypoxia (Fan et al., 2014).

Besides increasing NAD(P)H production, one-carbon flux induced by these oxygen and redox sensing pathways also contributes to the maintenance of the oxidative balance through generation of glutathione (Locasale, 2013; Lu et al., 2015). Glutathione is a tripeptide of glutamate, glycine, and cysteine and is a powerful antioxidant molecule (Ballatori et al., 2009). The increased serine production and catabolism induced by HIF1 and NRF2 leads to an increase in glycine as a byproduct of serine catabolism. It also contributes to an increase in cysteine which is a product of the trans-sulfuration pathway, thus resulting in higher levels of glutathione, facilitating rapid ROS detoxification (DeNicola et al., 2015).

This evidence asserts that not only are one-carbon units important to maintain redox balance, but also that the master regulators of redox remodeling participate in regulating flux through one-carbon metabolism ensuring a feedback mechanism that keeps homeostasis (Figure 2).

## One-carbon metabolism at the root of carcinogenesis

The indispensability of one-carbon metabolism in carcinogenesis is well demonstrated by many established cancer therapies. For decades, methotrexate has been used as a standard of care treatment for cancer patients (Newman and Maddocks, 2017). Methotrexate is in the class of drugs called anti-folates and is an inhibitor of DHFR, which prevents THF production and halts the folate cycle (Osborn et al., 1958). Another commonly used anti-cancer treatment, 5-fluorouracil (5-FU), is known to inhibit thymidylate synthase (TYMS), which catalyzes the transfer of a one-carbon unit from methylene-THF onto dUMP to make dTMP (Longley et al., 2003). The efficacy of methotrexate and 5-FU in the clinic demonstrates that many cancers are dependent on one-carbon metabolism.

More recently, it has been established that the repression of tumor suppressor genes by methylation, which is dependent on SAM levels, is a key-initiating event for many cancers (Kulis and Esteller, 2010). Oncogenic KRAS mutations have been shown to increase regional DNA methylation due to increased SAM obtained via one-carbon metabolism, resulting in increased tumor growth (Kottakis et al., 2016). *De novo* serine synthesis has also been identified as a metabolic vulnerability of many cancers, including non-small cell lung cancer (NSCLC), breast cancer, and melanoma (Locasale et al., 2011; Possemato et al., 2011; DeNicola et al., 2015). Increased expression of the enzymes involved in the SSP is frequently observed in these types of cancers, and overexpression of PHGDH in non-tumorigenic breast cells is sufficient to develop a cancerous phenotype (Locasale et al., 2011). Chemical and genetic inhibition of the SSP is sufficient to abrogate proliferation of cancer cells in culture and to reduce xenograft tumor burden (Possemato et al., 2011; Mullarky et al., 2016; Pacold et al., 2016). Additionally, increased activity of SHMT, has been observed in multiple cancers and shown to be critical for tumor formation (Jain et al., 2012; Ye et al., 2014). In fact, in the absence of SHMT and the SSP, tumors become addicted to exogenous serine and serine starvation reduces cancer cell proliferation and tumor growth (Maddocks et al., 2013, 2016; Labuschagne et al., 2014).

Glycine metabolism has also been shown to be important in cancer development and growth. The main enzyme in the glycine cleavage pathway, GLDC, is upregulated in lung-tumor initiating cells as well as glioblastomas, and sustained GLDC hyperactivity has been shown to be critical for tumorigenesis (Zhang et al., 2012). In a mouse embryonic fibroblast cell line, overexpression of GLDC alone was sufficient to induce tumorigenesis (Zhang et al., 2012). There is evidence suggesting that the GCS supports tumor survival by reducing toxicity due to accumulation of glycine (Kim et al., 2015). When there is an excess of glycine in a cell, such as when SHMT is rapidly converting serine to glycine, this alternative metabolic pathway becomes active and subverts the toxic accumulation of glycine while producing one carbon units (Kim et al., 2015). This evidence implicates glycine catabolism as a driver of carcinogenesis both by driving one-carbon metabolism and protecting from glycine toxicity. Additionally, interfering with the mitochondrial folate cycle by suppression of MTHFD1L also has a potent anti-tumor effect, further supporting the essential nature of the mitochondrial folate cycle for carcinogenesis (Ducker et al., 2016; Lee et al., 2017). In this scenario, it is possible that the lack of redundancy between the cytosolic and mitochondrial folate pathways is linked to hyperactivity of the GCS, which is solely mitochondrial.

## Oncogenes hijack sensing mechanisms to sustain one-carbon metabolism

In many cancer types, proto-oncogenes have their function altered, thus contributing to hijacking of the regulatory mechanisms that preserve homeostasis in healthy cells (Gomes and Blenis, 2015). Given that one-carbon metabolism is regulated by nutrient, energy, oxygen, and redox sensors working concertedly to keep homeostasis, the idea that oncogenes or loss of tumor suppressors manipulate one-carbon flux in tumorigenesis is attractive.

In support of this idea, the oncogene MYC has been shown to hijack regulatory pathways to increase flux through one-carbon metabolism in different cancers. For example, C-MYC induces SSP activity under nutrient deficient conditions in liver carcinomas (Sun et al., 2015). In neuroblastoma, N-MYC causes a HIF1-dependent induction of SHMT2 under hypoxic conditions (Ye et al., 2014). Additionally, loss of function mutations in the tumor suppressor KEAP1 have been shown to power one-carbon metabolism in NSCLCs (DeNicola et al., 2015). KEAP1 is the suppressor of NRF2. When its function is lost, a NRF2-induced upregulation of ATF4 triggers the SSP (Kansanen et al., 2013).

Oncogenic mutations promoting constitutive KRAS activation, one of the most common occurrences in cancer, correlates with increased expression of folate cycle enzymes (Moran et al., 2014). Oncogenic KRAS leads to aberrant activation of mTOR, which regulates both the SSP and the folate cycle (Gomes and Blenis, 2015; Ben-Sahra et al., 2016). Loss of another tumor suppressor, the AMPK activator liver kinase B1 (LKB1), is prevalent in KRAS mutant tumors (Kottakis et al., 2016). This suggests that along with stimulating mTOR, KRAS may also increase one-carbon metabolism through subversion of AMPK's inhibitory effects in the folate cycle.

The tumor suppressor p53 is also known to play a key role in one-carbon metabolism regulation. p53 is responsible for halting the cell cycle in conditions of stress, preventing uncontrolled proliferation (Maddocks et al., 2013). Loss of p53 is common in many types of cancer, conveying a survival advantage by allowing carcinogenic cells to replicate regardless of stress (Kruiswijk et al., 2015). Activation of p53 by non-genotoxic stresses in non-cancerous cells represses the expression of, PHGDH, to promote apoptosis (Ou et al., 2015). In tumors, loss of p53 causes addiction to serine (Maddocks et al., 2013). Consequently, serine starvation has been shown to considerably decrease growth of these tumors (Maddocks et al., 2013). So, it is interesting that, in cancers where p53 remains active, serine starvation leads to activation of p53, triggering cell cycle arrest. This phenomenon allows cells to channel serine into glutathione synthesis rather than the production of building blocks, therefore allowing cell survival (Maddocks et al., 2013; Kruiswijk et al., 2015). These paradoxical roles of p53 in regulating serine synthesis demonstrate the complexity of one-carbon metabolism regulation and the role it plays in different physiological conditions.

It is notable that the majority of evidence regarding oncogenic regulation of one-carbon metabolism lies in the upregulation of *de novo* serine synthesis. Upregulation of the SSP allows for flexibility to fuel various downstream pathways. Additionally, this phenomenon can be explained by the Warburg effect, where glucose consumption and oxidation become dysregulated to allow for rapid growth and proliferation (Liberti and Locasale, 2016). An increase in glycolysis leads to the accumulation of its intermediates, including the precursor for *de novo* serine synthesis, 3PG. By upregulating the SSP and other enzymes necessary for one-carbon metabolism, glucose-derived carbon can be shunted to a process capable of producing a variety of biomolecules and redox species (Lunt and Vander Heiden, 2011). This, along with upregulation of other glucose-derived pathways, is key for tumor growth and proliferation.

These examples demonstrate that activation and/or loss of oncogenes and tumor suppressors override the control of sensing mechanisms and drive flux through one-carbon metabolism, allowing tumors to thrive (Figure 2).

## Conclusions

As a growing body of evidence supports the key role of one-carbon metabolism in cancer, it becomes of interest to expand our knowledge on how one-carbon metabolism is regulated. Here, we propose that one-carbon metabolism integrates the nutrient, energetic, and redox statuses of cells, and that flux through associated pathways is fine-tuned to reflect said status and to ensure cellular homeostasis. Taking into consideration the critical role of one-carbon metabolism as a producer of reducing power and building blocks, as well as its part in regulating substrates for epigenetic and post-translational modifications, an important line of questioning emerges. What determines preferential utilization of the mitochondrial vs. the cytosolic folate cycle? Can we take advantage of one-carbon metabolism for the development of more efficacious cancer therapies? Additionally, can we use one-carbon metabolism as a predictor of responsiveness to chemotherapies such as methotrexate and 5-FU? Is one-carbon metabolism and its remarkable flexibility responsible for the development of drug resistance? The answers to these questions are still largely unknown but may prove vital for advances in precision medicine and the treatment of cancer.

## Author contributions

APG conceived, wrote and edited the manuscript. AR wrote and JB edited the manuscript.

### Conflict of interest statement

The authors declare that the research was conducted in the absence of any commercial or financial relationships that could be construed as a potential conflict of interest.
